# *Eucalyptus globulus* oil supplementation improves growth performance by regulating serum biochemistry, antioxidant, intestinal health, and lung health of broilers from 1 to 21 days of age

**DOI:** 10.3389/fvets.2026.1798261

**Published:** 2026-03-17

**Authors:** Zhenzhen Ji, Zhiqiang Miao, Yuanyang Dong, Miaomiao Han, Chenxuan Huang, Jianhui Li

**Affiliations:** 1 Laboratory of Poultry Production, College of Animal Science, Shanxi Agricultural University, Jinzhong, China; 2 College of Animal Science & Technology, Henan University of Animal Husbandry and Economy, Zhengzhou, China

**Keywords:** antioxidant, barrier function, broilers, *Eucalyptus globulus* oil, growth performance, immunity, serum biochemistry

## Abstract

**Introduction:**

*Eucalyptus globulus* oil (EGO) is distinguished by its elevated 1,8-cineole content and is gaining attention as a natural feed additive due to its multifunctional bioactivities. However, the specific impacts of EGO on broiler physiology, including serum biochemical parameters and organ barrier functions, are not yet fully understood. The objective of this research was to ascertain the impact of EGO on the growth of broiler chickens. This investigation encompassed various parameters, including serum biochemistry, antioxidant capacity, intestinal and lung immunological function, and barrier function aspects.

**Methods:**

A total of 288 one-day-old male Cobb broilers with similar weight (45.18 ± 1.01 g) were randomly divided into four groups of six replicates of 12 birds each. The control group (CON) was fed a basal diet, while the other three groups received a basal diet supplemented with 100, 200, or 300 mg/kg of EGO. The trial lasted 21 days. Performance indices, including average daily gain (ADG), average daily feed intake (ADFI), and feed-to-gain ratio (F/G), were evaluated at 7, 14, and 21 days of age. One bird per replicate was euthanized to collect blood, immune organ, lung, and jejunal tissue samples for further analysis at 21 days of age.

**Results:**

This supplementation increased ADG by 3.90% and decreased F/G by 3.97% (*p* < 0.05). It was evident that both ADG and F/G demonstrated a quadratic response to elevated EGO levels (*p* < 0.05). Serum analysis revealed linear and quadratic increases in alkaline phosphatase (ALP) and creatinine (Cr) levels with higher EGO doses (*p* < 0.05), while lysozyme (LZM) activity increased linearly (*p* < 0.05). The antioxidant capacity was enhanced, showing linear and quadratic improvements in superoxide dismutase (SOD) and glutathione peroxidase (GSH-Px) activities, and a reduction in malondialdehyde (MDA) (*p* < 0.05). In the jejunum, EGO was observed to down-regulate pro-inflammatory cytokines interleukin-1β (*IL-1β*), tumor necrosis factor-*α* (*TNF-α*), and interferon-*γ* (*IFN-γ*), and up-regulate the anti-inflammatory cytokine interleukin-10 (*IL-10*) (*p* < 0.05). Concurrently, EGO increased the expression of tight junction proteins occludin (*OCLN*), claudin-1 (*CLDN1*), tight junction protein 1 (*TJP1*), and mucin 2 (*MUC2*) (*p* < 0.05). In contrast, EGO has been demonstrated to up-regulate avian beta-defensin 5 (*AvBD5*), *IFN-γ*, and *IL-10* in lung tissue (*p* < 0.05). Furthermore, EGO has been shown to enhance *CLDN1* and *TJP1* expression (*p* < 0.05), while significantly reducing *OCLN* levels (*p* < 0.05).

**Conclusion:**

These findings suggest that, given growth performance, safety, and efficacy, an additive dosage of 200 mg/kg is recommended.

## 1 Introduction

In the 21st century, one of the greatest threats to global public health has been identified as the rapid spread of antibiotic resistance genes, driven by widespread antibiotic pollution (1). In livestock and poultry production, the overuse of antibiotics as growth promoters has accelerated this crisis. To address this issue, a ban on the use of growth-promoting veterinary antibiotics in feed was implemented by the Chinese Ministry of Agriculture and Rural Affairs as of 2020 (2). This policy aims to curb antimicrobial resistance. However, it has also caused significant challenges to the livestock industry. This has created an urgent need for effective antibiotic alternatives (3, 4). The use of plant-based feed additives, especially those containing bioactive compounds with antibacterial, antioxidant, and anti-inflammatory properties, has become one of the most promising and potential alternatives to antibiotics (5–7).

*Eucalyptus* essential oils are primarily derived through the steam or hydrodistillation of leaves. Volatile monoterpenoids are the main constituents of these oils, with 1,8-cineole (eucalyptol) being the predominant bioactive constituent (8). *Eucalyptus* species, particularly *E. globulus* and its main constituent, 1,8-cineole (eucalyptol), have a long history of traditional use in the treatment of respiratory ailments (9, 10). *E. globulus* oil (EGO) is commonly used as an expectorant to alleviate symptoms associated with respiratory conditions such as bronchitis and asthma, as seen in products like GeloMyrtol^®^ and GeloMyrtol forte^®^.

Modern mechanistic studies have elucidated the immunomodulatory properties of EGO and eucalyptol in the respiratory system. They have been shown to directly modulate pulmonary immune responses by suppressing inflammatory signaling pathways (NF-κB, MAPKs) in lung macrophages and downregulating pattern recognition receptors such as TREM-1 and NLRP3 (11). Additionally, eucalyptol influences respiratory tract immunity in a dose-dependent manner through modulation of CD8^+^ T cells, alveolar macrophage function, and immunoglobulin levels (12). In LPS-induced mouse monocyte-macrophages, eucalyptol reduces the production of inflammatory mediators (TNF-*α*, IL-6, NO, iNOS) by inhibiting the NF-κB/PKC and ERK1/2/p38 pathways (13). These findings establish a strong mechanistic basis for the respiratory benefits of EGO.

Despite extensive research on plant essential oils in poultry production, studies have predominantly focused on growth performance, antioxidant status, and intestinal health (14–16), with limited attention to the respiratory system. This is an especially significant discrepancy in the case of broilers, given the well-documented respiratory benefits of EGO in animal models. Recent advances have confirmed the existence of the gut-lung axis, which is a bidirectional communication pathway linking the intestinal tract and the respiratory system (17). This connection is rooted in their common embryonic origin and shared mucosal immune system, which enables crosstalk between these two distant organs (18–20). Consequently, interventions that modulate gut health may indirectly affect pulmonary immunity.

Previous studies have demonstrated that dietary EGO supplementation can positively influence gut health in animals. Fathi et al. (21) reported improved intestinal microbial balance and immune responses in rabbits fed EGO, while Mohebodini et al. (22) found that EGO enhanced growth performance and partially modulated cecal microflora in broiler chickens. However, to the best of our knowledge, there has been no comprehensive evaluation of the effects of dietary EGO on intestinal and respiratory immune and barrier functions in broilers within a single experimental framework.

In consideration of the well-documented respiratory health benefits of EGO, and the growing knowledge of the gut-lung axis, we hypothesized that dietary EGO supplementation may produce positive effects on both intestinal and lung health through this bidirectional communication pathway. Specifically, EGO may enhance immune and barrier functions in the intestines and lungs while improving systemic antioxidant capacity, ultimately promoting growth performance. Therefore, this study aimed to investigate whether EGO could serve as an effective antibiotic alternative by supporting both intestinal and lung health in broiler chickens.

## 2 Materials and methods

### 2.1 Experimental materials

One-day-old male Cobb broiler chickens were procured from a livestock company in Henan Province. The pure *Eucalyptus globulus* oil (containing 86.96% 1,8-cineole, 9.10% limonene, 0.89% p-cymene, 0.79% *γ*-terpinene, and 0.57% myrcene) was procured from a company specializing in flavor and fragrance company in Yunnan Province. To ensure homogeneous distribution in the diets, pure *Eucalyptus globulus* oil was mixed with carrier (silicon dioxide) at a 1:6 ratio. The mixture was then spray-dried to form a powder.

### 2.2 Experimental design and management

A total of 288 one-day-old male Cobb broilers with similar weight (45.18 ± 1.01 g) were randomly divided into four groups of six replicates of 12 birds each. The control group (CON) was fed a basal diet, while the other three groups received a basal diet supplemented with 100, 200, or 300 mg/kg of the mixture product of *Eucalyptus globulus* oil (EGO). The doses of EGO were selected based on recent literature (23) and standard dose–response design principles. The trial lasted 21 days. The basal diet (Table 1) was a corn-soybean meal prepared according to the NRC (1994) standard and the “Nutrition Requirements of Chinese Chicken (NY/T 33-2004)” (issued by the Ministry of Agriculture). Each replicate of 12 broiler chickens was housed in each metal cage (70 × 54 × 40 cm). The temperature in the chicken house was kept between 32 °C and 34 °C in the first week after hatching, decreasing by 2–3 °C every other week until the final temperature of 24 °C was reached. Lighting conditions followed the standard of 23 h of light and 1 h of darkness. The broiler chickens were fed manually on two occasions per day, with ad libitum access to food and water. The same environmental, sanitary, and management conditions were maintained in broiler housing throughout the three-week experimental period. Vaccination was performed in accordance with the “National Animal Disease Immunization Technical Guidelines for 2023” (issued by the China Animal Disease Prevention and Control Center), with chickens receiving an inactivated Newcastle disease vaccine at 7 days of age.

**Table 1:** Composition and nutrient levels of the basal diet (air-dry basis).

Ingredients	Content, %	Nutrient content	Content^b^
Corn	57.40	Metabolizable energy, MJ·kg^−1^	12.37
Soybean meal	33.00	Crude protein, %	22.21
Fish meal	3.00	Calcium, %	1.04
Soybean oil	2.50	Total phosphorus, %	0.76
Calcium hydrogen phosphate	1.60	Available phosphorus, %	0.54
Limestone	1.20	Lysine, %	1.30
L-Lysine	0.05	Methionine, %	0.61
DL-Methionine	0.25	Methionine + Cystine, %	0.98
Salt	0.30	Tryptophan, %	0.26
Premix^a^	0.70	Threonine, %	0.84
Total	100.00		

a The premix provided per kilogram of diet: vitamin A:12,000 IU, vitamin D3:6,000 IU, vitamin E:50 IU, vitamin K3:1 mg, vitamin B1:3 mg, vitamin B2:8 mg, vitamin B6:7.5 mg, vitamin B12:0.6 mg, folic Acid:2 mg, pantothenic Acid:12 mg, nicotinamide:50 mg, copper:10 mg, zinc:80 mg, iron:50 mg, manganese:80 mg, selenium:0.30 mg, iodine:0.4 mg.

b Calculated value.

### 2.3 Sample collection

At 21 days of age and following a 12-h fast, one broiler with a body weight close to the replicate mean (6 birds per treatment) was selected for blood sampling from the wing vein. The blood samples were left at ambient temperature for 30 min. Following this, they were subjected to a centrifugal process at a speed of 3,000 revolutions per minute for a duration of 15 min, with the objective of separating of the serum. Subsequently, the serum was stored at −20 °C for the purpose of analysis. Following the collection of blood samples, the broilers were euthanised by cervical dislocation. Subsequently, left lung and jejunum tissues were collected and cryopreserved at −80 °C for subsequent analysis.

### 2.4 Growth performance

Growth performance data were collected and analyzed for accumulated periods of 1 to 7, 8 to 14, 15 to 21, and 1 to 21 days of age. All broilers were weighed separately on the 7th, 14th, and 21st days of the trial period, after fasting for 4 h. The amount of feed added and the remaining feed quantity were recorded for each replicate in each trial period. The average daily gain (ADG), average daily feed intake (ADFI), and feed-to-gain ratio (F/G) were measured.

### 2.5 Serum biochemical parameters

Total bilirubin (TBIL), total protein (TP), albumin (ALB), globulin (GLO), aspartate aminotransferase (AST), alkaline phosphatase (ALP), glucose (GLU), total cholesterol (TC), triglycerides (TG), high-density lipoprotein (HDL), low-density lipoprotein (LDL), creatinine (Cr), urea (UREA), calcium (Ca), phosphorus (P) in serum were measured using a fully automated biochemical analyzer (ADVIA XPT, SIEMENS, Tokyo, Japan). Serum lysozyme (LZM) was measured by turbidimetric assay using a kit purchased from Nanjing Jiancheng Biotechnology Institute. The albumin-to-globulin ratio (A/G) was also calculated.

### 2.6 Antioxidant parameters

The serum malondialdehyde (MDA) content, as well as the activities of superoxide dismutase (SOD), glutathione peroxidase (GSH-Px), and catalase (CAT), were determined by ELISA. The MDA (BC0025), CAT (BC0205), SOD (BC5165), and GSH-Px (BC1195) kits were purchased from Beijing Solare Technology Co., Ltd. (Beijing, China).

### 2.7 Immune organ indices

The spleen, thymus, and bursa of Fabricius were collected and weighed. The organs were then blotted dry with filter paper, and the organ index was calculated as follows: Organ index (g/kg) = organ weight/live weight.

### 2.8 Gene expression analysis

30–50 mg of lung and jejunal tissue samples were placed in 2 mL centrifuge tubes, to which 1 mL of tissue lysate was added, and low-temperature homogenization was carried out using a homogenizer (Freeze Milling Instrument JXFSTPRP-CLN, Shanghai Jingshun). The RNA was extracted using Total RNA Extraction Kit (DP419, TianGen Biochemical Technology Co., Ltd., Beijing, China) and its concentration was measured using an Ultra-Micro Spectrophotometer (Thermo Scientific NanoDrop™ One/One c, Waltham, United States).

The samples were reverse transcribed using the Novozymes HiScript III All-in-One RT SuperMix Perfect for qPCR (R333-01) reverse transcription kit. Reverse transcription was performed using an Applied Biosystems SimpliAmp PCR instrument (Thermo Scientific). The reverse transcription reaction program was set to 50 °C for 15 min and 85 °C for 5 s. Ct values were analyzed for all genes (see Table 2 for Gene Primer Sequence List) using the 2^−∆∆Ct^ method with GAPDH as an endogenous control.

**Table 2:** Sequence list of gene primers.

Gene	Gene name	Primer sequence (5′–3′)	Gene bank no.
*GAPDH*	Glyceraldehyde-3 phosphate dehydrogenase	F: GCTAAGGCTGTGGGGAAAGT R: TCAGCAGCAGCCTTCACTAC	NM_204305.2
*AvBD5*	Avian beta-defensin 5	F: ATGCAGATCCTGACTCTCCTCT R: TCAGGAATACCATCGGCTCCGG	NM_001001608.2
*IL-1β*	Interleukin-1β	F: GCTCAACATTGCGCTGTACC R: AGGCGGTAGAAGATGAAGCG	FJ537850.1
*IL-6*	Interleukin-6	F: ACGAGGAGAAATGCCTGACG R: CTTCAGATTGGCGAGGAGGG	NM_204628.2
*IL-10*	Interleukin-10	F: TGCGAGAAGAGGAGCAAAGC R: AACTCCCCCATGGCTTTGTAG	AJ621254.1
*TNF-α*	Tumor necrosis factor-α	F: CCCATCCCTGGTCCGTAAC R: CGGCGGCGTATACGAAGTA	MF000729
*IFN-γ*	Interferon-γ	F: CTGACAAGTCAAAGCCGCAC R: CTTCACGCCATCAGGAAGGT	NM_205149.2
*CLDN1*	Claudin 1	F: CTGGGTCTGGTTGGTGTGTT R: CGAGCCACTCTGTTGCCATA	NM_001013611.2
*TJP1*	Tight junction protein 1	F: TATGAAGATCGTGCGCCTCC R: GAGGTCTGCCATCGTAGCTC	XM_015278977
*OCLN*	Occludin	F: TACATCATGGGCGTCAACCC R: CCAGATCTTACTGCGCGTCT	NM_205128.1
*MUC2*	Mucin 2	F: AATGCTGAGTTCTTGCCTAA R: TGTTGCAGTTCATATCCTGGT	XM_001234581.3
*MUC5AC*	Mucin 5 AC	F: AAGACGGCATTTATTTCTCCAC R: TCATTACCAACAAGCCAGTGA	XM_003641322.2

### 2.9 Data analysis

The data were preprocessed using Excel 2013. The data were analyzed using a one-way ANOVA procedure in IBM SPSS Statistics version 24. Duncan’s test was then used for multiple-group comparisons. Linear and quadratic regression models were applied to analyze the relationships between EGO levels and the measured parameters. The results are expressed as the mean ± SEM, with *p* < 0.05 being considered statistically significant. Figures were generated using Origin 2021. The schematic graphics was drawn on https://biogdp.com.

## 3 Results

### 3.1 Growth performance

The effects of different concentrations of EGO on broiler growth performance are presented in Table 3. No broiler deaths occurred throughout the entire trial period. As shown in Table 3, there were no significant differences in ADG or F/G between the four groups on days 1–7 or 8–14 of age (*p* > 0.05), and ADFI was unchanged across all phases (*p* > 0.05). From days 15 to 21 of age, compared with the CON group, ADG in the 200 mg/kg EGO group was increased by 5.23% (*p* < 0.05); in contrast, F/G was decreased by 6.82% (*p* < 0.05). In the period of days 1–21 of age, the respective increases and decreases were 3.90 and 3.97% (*p* < 0.05). As the level of EGO supplementation in the diet increased, ADG first increased and then decreased, whereas F/G first decreased and then increased; both ADG and F/G showed a quadratic change at days 1–21 of age (*p* < 0.05).

**Table 3:** Effects of different concentrations of EGO on the growth performance of broilers (*n* = 6).

Items	CON	EGO (mg/kg)			SEM	*p*-value		
		100	200	300		Deal with	Linear	Quadratic
Day 1–7								
ADG (g/d)	20.16	20.45	20.70	20.45	0.101	0.338	0.225	0.201
ADFI (g/d)	23.25	23.45	23.83	23.70	0.106	0.216	0.066	0.137
F/G (g/g)	1.15	1.15	1.15	1.16	0.004	0.869	0.624	0.699
Day 8–14								
ADG (g/d)	41.83	42.99	42.76	41.63	0.506	0.749	0.857	0.539
ADFI (g/d)	53.67	54.54	52.99	53.90	0.424	0.665	0.829	0.977
F/G (g/g)	1.29	1.27	1.24	1.30	0.012	0.371	0.988	0.348
Day 15–21								
ADG (g/d)	71.76^b^	71.98^b^	75.51^a^	69.67^b^	0.690	0.014	0.667	0.074
ADFI (g/d)	94.54	93.62	92.92	92.37	0.440	0.351	0.065	0.186
F/G (g/g)	1.32^a^	1.30^ab^	1.23^b^	1.33^a^	0.014	0.063	0.697	0.140
Day 1–21								
ADG (g/d)	44.58^b^	45.14^ab^	46.32^a^	43.91^b^	0.300	0.021	0.768	0.037
ADFI (g/d)	57.15	57.20	56.58	56.66	0.200	0.606	0.247	0.520
F/G (g/g)	1.26^a^	1.24^ab^	1.21^b^	1.26^a^	0.007	0.028	0.880	0.042

^a,b^ In the same row, values with different letter superscripts indicate significant differences (*p* < 0.05). SEM = total standard error of means. CON group: fed a basal diet.

### 3.2 Serum biochemical parameters

As presented in Table 4, the supplementation of the diet with 300 mg/kg of EGO resulted in a significantly higher serum ALP activity in broilers in comparison to in the CON group, with an increase of 45.24% (*p* < 0.05). In the 300 mg/kg EGO group, broilers exhibited significantly elevated serum Cr levels in comparison to the CON group, demonstrating a 68.37% increase (*p* < 0.05). As the level of EGO added to the diet increased, broilers showed linear (*p* < 0.05) and quadratic (*p* < 0.05) changes in ALP activity and Cr levels, as well as a linear (*p* < 0.05) increase in LZM activity.

**Table 4:** Effects of different concentrations of EGO on serum biochemical parameters of broilers (*n* = 6).

Items	CON	EGO (mg/kg)			SEM	*P*-value		
		100	200	300		Deal with	Linear	Quadratic
TBIL (μmol/L)	2.95	3.13	3.06	1.75	0.440	0.667	0.342	0.461
TP (g/L)	31.77	37.00	34.34	34.78	1.218	0.516	0.558	0.505
ALB (g/L)	4.43	6.33	6.26	6.07	0.523	0.546	0.310	0.362
GLO (g/L)	27.33	30.67	28.08	28.72	0.755	0.454	0.806	0.624
A/G	0.17	0.21	0.21	0.21	0.012	0.627	0.317	0.420
AST (U/L)	318.50	357.50	218.80	404.00	33.755	0.294	0.654	0.578
ALP (U/L)	3197.33^b^	3630.50^ab^	3841.20^ab^	4643.83^a^	194.340	0.041	0.004	0.017
GLU (mmol/L)	11.97	11.06	8.72	10.58	0.664	0.420	0.297	0.365
TC (mmol/L)	3.32	3.82	4.03	3.95	0.158	0.400	0.140	0.221
TG (mmol/L)	0.57	1.45	1.32	1.27	0.276	0.686	0.430	0.511
HDL (mmol/L)	2.46	2.62	2.74	2.69	0.104	0.824	0.404	0.634
LDL (mmol/L)	2.09	2.55	2.63	2.57	0.110	0.287	0.120	0.148
Cr (μmol/L)	6.83^b^	8.05^b^	8.20^b^	11.50^a^	0.435	<0.001	<0.001	<0.001
UREA (mmol/L)	0.73	1.25	1.12	1.18	0.152	0.642	0.373	0.506
Ca (mmol/L)	1.55	1.56	1.60	1.57	0.017	0.725	0.460	0.637
P (mmol/L)	2.08	2.01	2.13	2.11	0.028	0.502	0.438	0.666
LZM (U/mL)	8.61	8.78	9.11	9.17	0.102	0.148	0.021	0.072

^a,b^ In the same row, values with different letter superscripts indicate significant differences (*p* < 0.05). SEM = total standard error of means. CON group: fed a basal diet.

### 3.3 Serum antioxidant parameters

As shown in Table 5, in comparison with the CON group, the addition of 200 and 300 mg/kg of EGO significantly increased serum SOD activity by 41.56 and 34.66%, respectively (*p* < 0.05). Meanwhile, it was demonstrated that a dose of 200 mg/kg of EGO enhanced serum CAT activity by 16.93% (*p* < 0.05). GSH-Px activity of broilers in 100, 200, and 300 mg/kg of EGO groups were 353.95, 427.17, and 523.63% higher than that in the CON group (*p* < 0.05). The addition of 300 mg/kg of EGO reduced serum MDA levels by 29.73% in comparison to the CON group (*p* < 0.05). The activity of SOD and GSH-Px, as well as MDA levels, showed linear and quadratic changes (*p* < 0.05) as the amount of EGO added to the diet increased. SOD activity showed an initial rise followed by a decline, but the turning points for GSH-Px and MDA have not yet appeared.

**Table 5:** Effects of different concentrations of EGO on serum antioxidant indicators of broilers (*n* = 6).

Items	CON	EGO (mg/kg)			SEM	*P*-value		
		100	200	300		Deal with	Linear	Quadratic
SOD (U/mL)	37.80^b^	37.05^b^	53.51^a^	50.90^a^	1.785	<0.001	<0.001	<0.001
CAT (U/mL)	24.99^b^	25.07^b^	29.22^a^	25.41^b^	0.620	0.047	0.379	0.245
GSH-Px (U/mL)	369.03^a^	1675.21^b^	1945.40^b^	2301.39^b^	193.542	<0.001	<0.001	<0.001
MDA (nmol/mL)	10.09^a^	8.19^ab^	8.00^ab^	7.09^b^	0.406	0.043	0.006	0.020

^a,b^ In the same row, values with different letter superscripts indicate significant differences (*p* < 0.05). SEM = total standard error of means. CON group: fed a basal diet.

### 3.4 Immune organ indices

As demonstrated in Table 6, the incorporation of specific levels of EGO into the diet did not demonstrate a substantial impact on the indices of the spleen, bursa of Fabricius, and thymus of broilers (*p* > 0.05). Nevertheless, an upward trend in spleen indexes was observed (*p* = 0.086). No linear or quadratic trends were observed (*p* > 0.05).

**Table 6:** Effects of different concentrations of EGO on immune organ indices of broilers (*n* = 6).

Items	CON	EGO (mg/kg)			SEM	*P*-value		
		100	200	300		Deal with	Linear	Quadratic
Spleen index (g/kg)	0.95	1.25	1.14	1.15	0.042	0.086	0.204	0.109
Bursa of Fabricius index (g/kg)	1.93	2.13	1.98	1.95	0.043	0.346	0.786	0.403
Thymus index (g/kg)	4.09	4.46	4.38	4.23	0.065	0.184	0.574	0.106

### 3.5 Expression of immune factors genes in jejunum and lung

**Figure 1:**
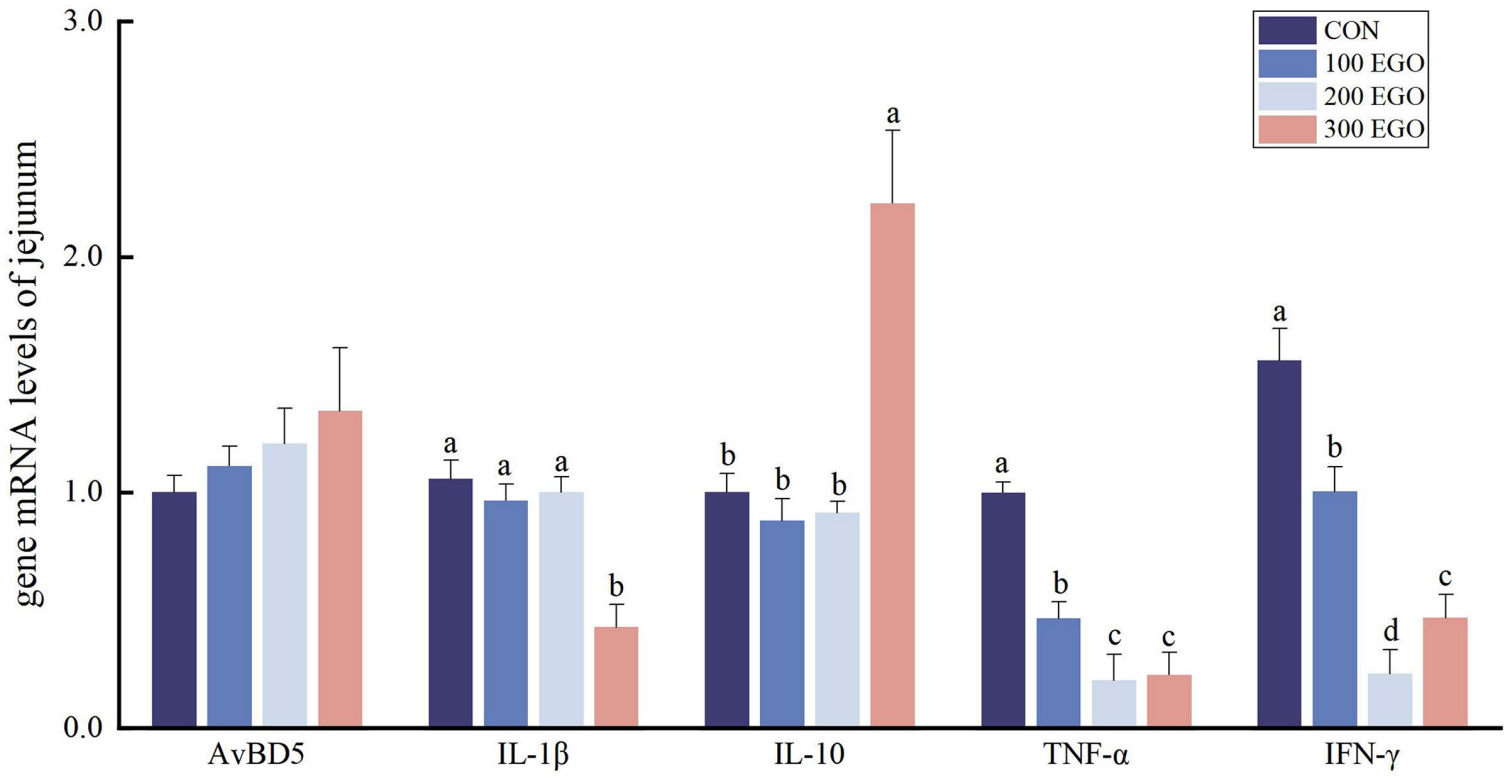
Effect of different concentrations of EGO on immune factor genes expression in the jejunum of broilers (*n* = 6). CON, basal diet group; 100 EGO, 200EGO, and 300EGO, basal diet supplemented with 100, 200, and 300 mg/kg of EGO, respectively. ^a-d^ In the same indicator, values with different letter superscripts indicate a significant difference (*p* < 0.05).

As demonstrated in Figure 1, no statistically significant variations in *AvBD5* expression level were observed in the jejunum of broiler chickens across the four experimental groups (*p* < 0.05). *IL-1β* expression level in the jejunum of broilers in the 300EGO group were significantly lower than those in the CON group by 59.53% (*p* < 0.05), while *IL-10* expression level were significantly higher by 122.31% (*p* < 0.05). Supplementing diets with 100, 200, and 300 mg/kg EGO significantly down-regulated *TNF-α* and *IFN-γ* expression in the jejunum by 53.50, 79.68, and 77.32%, and by 35.66, 85.14, and 69.97%, respectively (*p* < 0.05).

**Figure 2:**
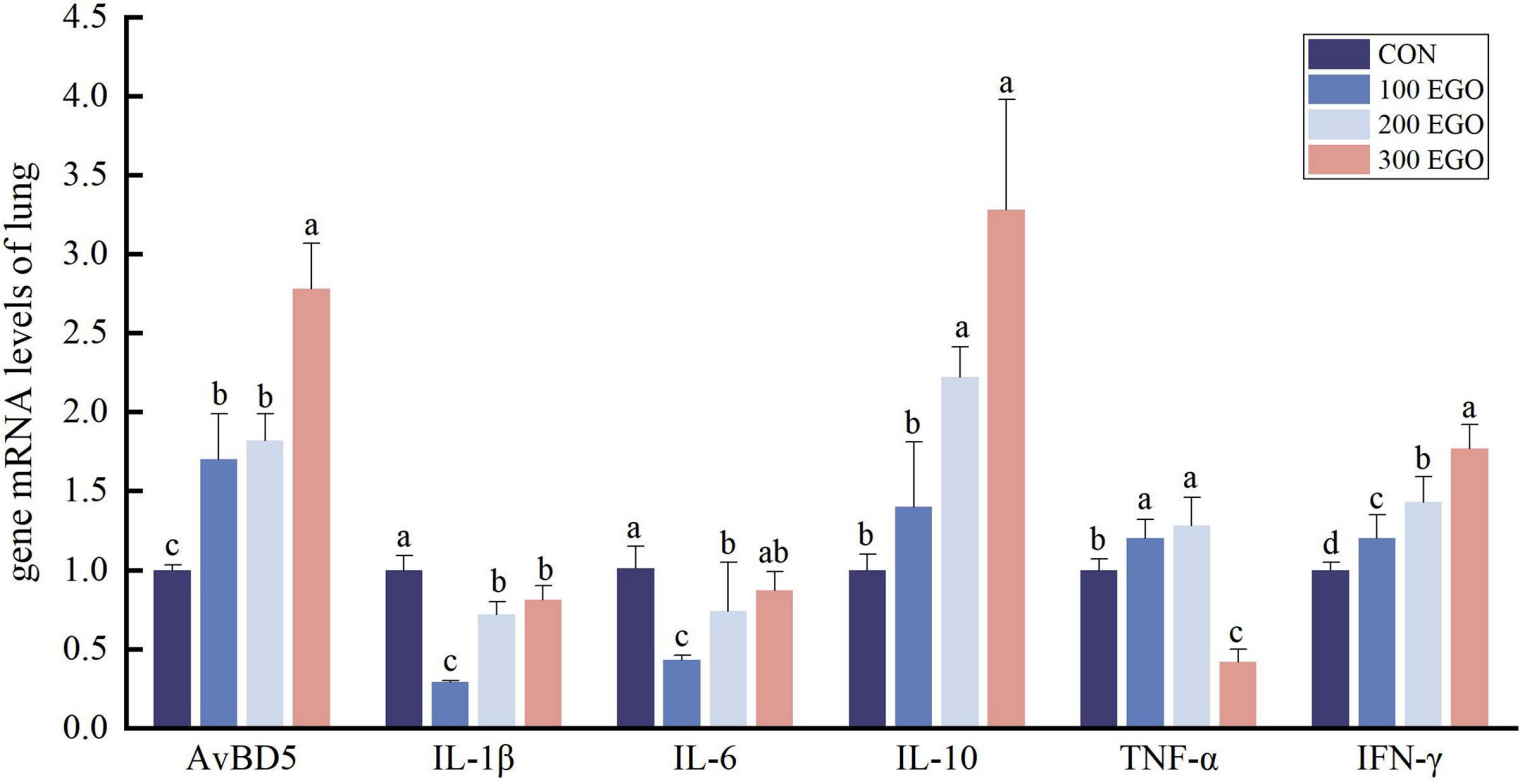
Effect of different concentrations of EGO on immune factor genes expression in lung tissue of broilers (*n* = 6).

As shown in Figure 2, dietary EGO at different levels significantly affected immune gene expression in broiler lung tissue. Compared with the CON group, 100, 200, and 300 mg/kg EGO up-regulated *AvBD5* and *IFN-γ* expression by 70.32, 82.04, and 178.06%, and by 20.09, 42.86, and 77.16%, respectively (*p* < 0.05), while down-regulated *IL-1β* expression by 71.46, 28.67, and 19.69%(*p* < 0.05). *IL-10* expression level in the 200EGO and 300EGO groups was increased by 121.11 and 227.08%, respectively (*p* < 0.05). For *IL-6*, 100 and 200 mg/kg EGO reduced expression by 56.91 and 26.23%(*p* < 0.05), whereas 300 mg/kg EGO decreased *TNF-α* expression level by 57.60% (*p* < 0.05). In contrast, 100 and 200 mg/kg EGO increased *TNF-α* expression by 20.24 and 27.82%, respectively (*p* < 0.05).

### 3.6 Expression of barrier function-associated genes in jejunum and lung

**Figure 3:**
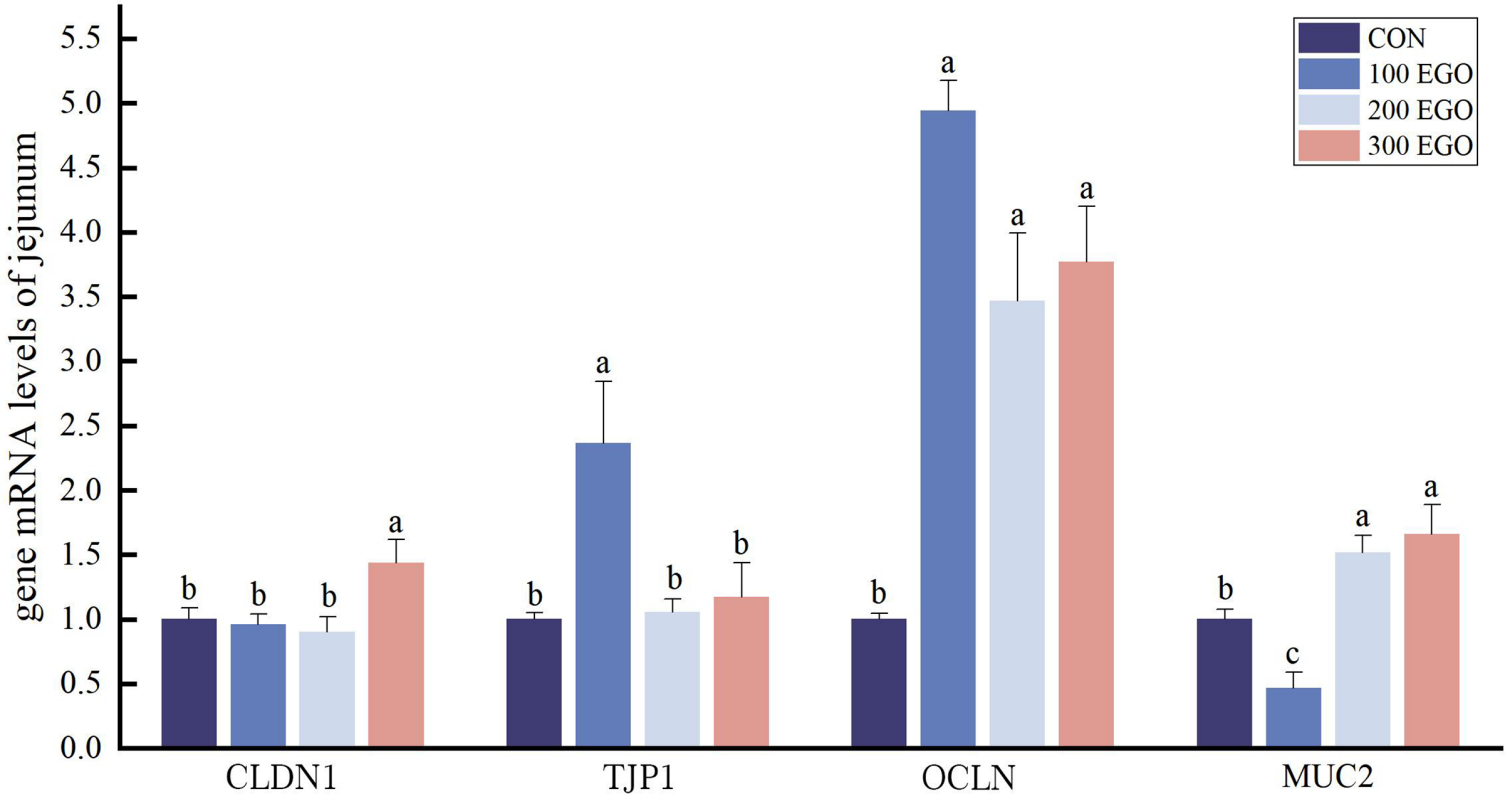
Effect of different concentrations of EGO on barrier function-associated gene expression in the jejunum of broilers (*n* = 6).

As shown in Figure 3, *CLDN1* expression was found to be 43.24% higher in the jejunum of broiler chickens in the 300EGO group than in the CON group (*p* < 0.05). While *TJP1* expression was increased by 136.40% in the jejunum of broiler chickens in the 100EGO group (*p* < 0.05). *OCLN* expression level in the jejunum in the 100EGO, 200EGO, and 300EGO groups were significantly up-regulated by 393.95, 246.76, and 276.52%, respectively (*p* < 0.05). Compared with the CON group, *MUC2* expression in the jejunum was significantly up-regulated by 51.61 and 65.66% in the 200 EGO and 300 EGO groups, respectively (*p* < 0.05), but significantly down-regulated by 53.15% in the 100 EGO group (*p* < 0.05).

**Figure 4:**
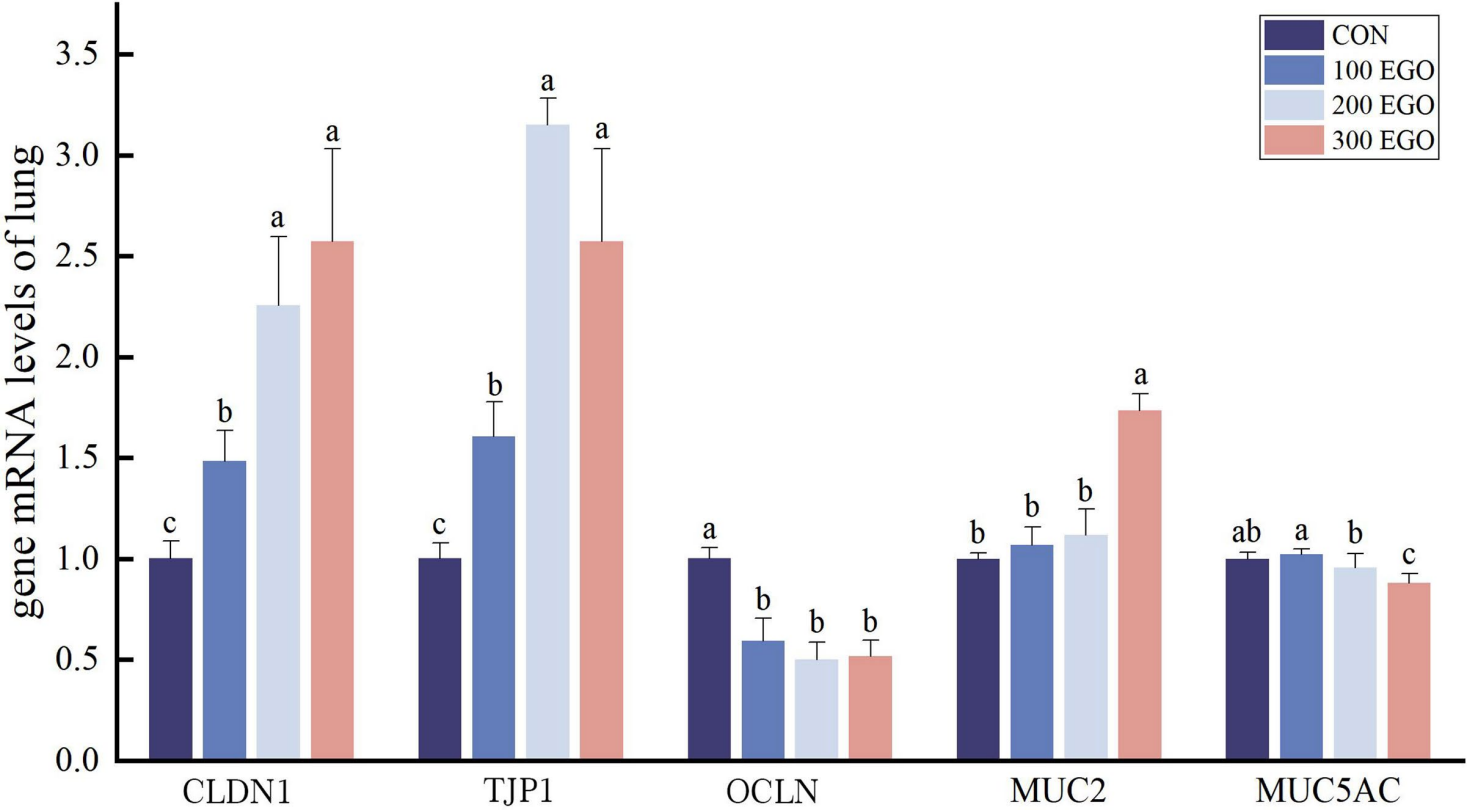
Effect of different concentrations of EGO on barrier function-associated gene expression in the lung tissue of broilers (*n* = 6).

As shown in Figure 4, *CLDN1* and *TJP1* expression in the lungs of broilers in the 100EGO, 200EGO, and 300EGO groups were increased significantly by 48.13, 125.18, and 156.61%, respectively, and by 60.24, 214.31, and 156.76%, respectively (*p* < 0.05). However, *OCLN* expression level decreased significantly by 40.57, 49.88, and 48.25% (*p* < 0.05). The *MUC2* expression level of the 300EGO group was 73.63% higher than that in the CON group (*p* < 0.05). However, the *MUC5AC* expression level in the 200EGO and 300EGO groups was 4.49 and 11.82% lower, respectively (*p* < 0.05).

## 4 Discussion

Plant essential oils with their antioxidant, antimicrobial, anti-inflammatory, and growth-promoting properties are increasingly regarded as promising alternatives to antibiotics (24, 25). However, research on EGO in livestock production remains relatively limited, with most studies focusing on blended essential oils or other single-source oils containing 1,8-cineole. The growth-promoting effects of EGO are primarily attributed to its major bioactive constituent, 1,8-cineole (eucalyptol). Several studies have demonstrated that dietary or water supplementation with 1,8-cineole or eucalyptus essential oil improves ADG and reduces F/G in broilers, although the effective dose ranges vary considerably across studies (21, 23, 26). Consistent with these findings, the present study found that 200 mg/kg EGO significantly enhanced ADG and reduced F/G during both the days 15–21 of age and the overall days 1–21 of age periods, without affecting ADFI. The enhancement in growth performance, in the absence of a concomitant increase in feed intake, indicates that EGO does not merely stimulate appetite, but rather enhances nutrient utilization efficiency. The improvement in growth performance without a concomitant increase in feed intake suggests that EGO enhances nutrient utilization efficiency rather than simply stimulating appetite. This effect may be mediated through multiple mechanisms. First, plant essential oils containing 1,8-cineole can improve intestinal morphology. For instance, Salehifar et al. (27) observed that Myrtaceae essential oils increased villus height in the jejunum and ileum and the thickness of intestinal epithelial cells in broiler chickens, thereby expanding the absorptive surface area for nutrients. Second, the antioxidant properties of 1,8-cineole may promote growth by reducing the metabolic costs associated with oxidative stress. Activation of the Nrf2 pathway by 1,8-cineole up-regulates endogenous antioxidant enzymes (e.g., CAT, SOD, GSH-Px), protecting intestinal epithelial cells from oxidative damage and maintaining optimal absorptive function (28). Third, the anti-inflammatory properties of 1,8-cineole, which are a consequence of the suppression of NF-κB and MAPK signaling, have the potential to reduce the low-grade systemic inflammation that is commonly associated with the rapid growth phases of broilers. This process would result in the redirection of energy from immune activation to tissue deposition (11). The results of this study demonstrate a dose-dependent relationship between EGO’s effects and dosage, with optimal outcomes observed at 200 mg/kg and diminished or adverse effects noted at 300 mg/kg. This finding aligns with the hormesis concept, which posits that low to moderate doses of bioactive compounds elicit beneficial effects, while higher doses may potentially induce adverse outcomes. This pattern is consistent with the results reported by Shao et al. (12), who established that eucalyptol regulates the immune system of the respiratory tract in a dose-dependent manner, where excessive doses may result in adverse effects. The critical turning point that was observed in the 200–300 mg/kg EGO dose range in this study underscores the paramount importance of meticulous dose optimization in practical applications. Thus, careful consideration of oil composition and supplementation levels is critical for practical applications.

Serum biochemical parameters reflect the physiological status of various systems in animals. Among these, ALP, an enzyme catalyzing the hydrolysis of phosphate monoesters, is closely associated with bone metabolism. Elevated ALP activity generally reflects enhanced osteoblast activity and bone formation, as it promotes collagen synthesis and bone salt deposition (29). In the present study, ALP activity remained unchanged at 100 and 200 mg/kg EGO but increased significantly at 300 mg/kg. The observed dose-dependent elevation in bone metabolism suggests that EGO supplementation at higher levels may promote enhanced skeletal development and contribute to improved overall growth, although further research is required to fully elucidate its mechanism of action and optimal dosages. This interpretation is consistent with the findings of previous reports, which demonstrated that eucalyptol enhances osteoblast differentiation through the process of ERK phosphorylation (30). However, it is crucial to exercise caution when interpreting the observed increase in ALP, as it may be indicative not only of enhanced bone metabolism, but also of a broader physiological response to high-dose EGO exposure. Serum Cr, a marker of renal filtration capacity, is widely used to assess kidney function. Elevated serum creatinine is indicative of a decrease in glomerular filtration rate (31). The substantial increase observed at 300 mg/kg raises a safety concern, suggesting a potential renal burden or altered renal hemodynamics at this high dose. Although other renal function markers (e.g., UREA) remained unchanged in this study, the isolated elevation in Cr suggests a need for caution. Although the 300 mg/kg EGO supplementation improved certain growth and antioxidant parameters, this dose cannot be recommended for practical application due to safety concerns. LZM, a critical component of innate immunity, is present in neutrophils, monocytes, macrophages, and mucosal secretions. It exerts antibacterial effects by degrading glycosidic bonds in pathogens such as *Escherichia coli* and Staphylococcus (32). Serum LZM activity serves as an indirect indicator of monocyte/macrophage functional status. In this study, EGO supplementation at 100–300 mg/kg tended to increase LZM activity, suggesting enhanced innate immune competence. This finding is consistent with those reported by Zhou et al. (33), who found that perilla essential oil significantly increased serum lysozyme levels in mice, thereby highlighting the immunomodulatory potential of plant essential oils. In summary, the administration of EGO supplementation at a dose of 200 mg/kg has been demonstrated to achieve a balance between efficacy and safety. The supplementation has been shown to enhance growth performance and innate immunity without compromising renal or hepatic function.

Redox reactions are of fundamental importance to an organism’s metabolism, with the process generating substantial quantities of free radicals. Healthy animals have been shown to maintain redox homeostasis by neutralizing free radicals through the utilization of their own antioxidant defense systems. It has been established that SOD, CAT, and GSH-Px can mitigate oxidative damage by scavenging reactive oxygen species (34). MDA, a byproduct of polyunsaturated fatty acid peroxidation, has been shown to serve as a reliable biomarker for lipid peroxidation (35). Plant essential oils are characterized by their abundance of phenolic and terpenoid compounds, which confers upon them intrinsic antioxidant properties. Consequently, there has been an increasing tendency for their incorporation into poultry feed additives, with a view to enhancing health and performance (36, 37). It has been demonstrated in previous studies that EGO and its primary constituent, 1,8-cineole, have the capacity to modulate antioxidant status in animals. For instance, Mohebodini et al. (21) reported that dietary EGO increased serum SOD activity and reduced MDA levels in broilers. *In vitro*, 1,8-cineole has been shown to protect rat pheochromocytoma cells from hydrogen peroxide-induced oxidative stress by up-regulating the expression of CAT, SOD, and GSH-Px while suppressing intracellular free radical generation (38). In accordance with these findings, the present study demonstrated that EGO supplementation (100–300 mg/kg) led to a significant increase in the activities of SOD, CAT, and GSH-Px, accompanied by a concurrent reduction in serum MDA levels. Collectively, these observations signify an enhanced systemic antioxidant capacity in broilers. The most striking observation was the dose-dependent increase in GSH-Px activity, which rose by 353.95, 427.17, and 523.63% in the 100, 200, and 300 mg/kg EGO groups, respectively. GSH-Px, as a key enzyme in the body’s antioxidant defense system, exhibits significantly increased activity that is typically attributed to the activation of the Nrf2 signaling pathway (39). The primary components of blue eucalyptus essential oil, 1,8-cineole and *α*-pinene, can induce Nrf2 nuclear translocation and scavenge reactive oxygen species, thereby inhibiting lipid peroxidation and enhancing the expression of the enzymatic antioxidant system (28). The substantial increase in antioxidant enzyme activity demonstrates that EGO displays considerable antioxidant activity, a finding corroborated by other antioxidant indicators in this study, including the decline in MDA content. The dose-dependent nature of the antioxidant response—with 300 mg/kg EGO producing the greatest enzyme induction—suggests that within the tested range, higher doses confer greater antioxidant protection. However, this must be balanced against the potential renal safety concerns identified at 300 mg/kg (elevated creatinine). The 200 mg/kg dose appears to offer substantial antioxidant enhancement without compromising safety, supporting its selection as the optimal practical dose.

The development of immune organs is closely linked to an organism’s overall immune status. The spleen functions as a reserve for mature lymphocytes, the thymus serves as a central hub for cellular immunity and the differentiation and maturation of T cells, and the bursa of Fabricius is a distinctive organ of the humoral immune system found in chickens The growth and function of these organs directly determine immune competence, and immune organ indices are a standard metric for evaluating immunity. In the present study, dietary supplementation with EGO (100–300 mg/kg) did not significantly affect the indices of spleen, thymus, or bursa of Fabricius in broilers. This absence of effect is consistent with findings reported by Qin et al. (26), who observed that 50–150 mg/kg eucalyptus essential oil similarly failed to alter immune organ development in broilers. However, these results contrast with those of Farhadi et al. (40), who reported that supplementing the basal diet with 250 mg/kg and 500 mg/kg of eucalyptus essential oil (more than 70% 1,8-cineole) increased antibody titers to vaccines in broiler chickens. The inconsistent outcomes may stem from variations in oil composition (e.g., 1,8-cineole content).

Inflammation represents a complex biological response of the immune system to antigenic stimuli, orchestrated by intricate interactions among immune cells, cytokines, and signaling pathways (41). Among these, pro-inflammatory cytokines—including TNF-*α*, IL-1*β*, IL-6, and IFN-*γ*—serve as key mediators of inflammatory responses, while anti-inflammatory cytokines such as IL-10 function to limit and resolve inflammation (42, 43). β-defensins, a class of antimicrobial peptides, also exhibit immunomodulatory properties (44). In the present study, dietary supplementation with EGO reduced the expression of pro-inflammatory cytokines (*IL-1β*, *TNF-α*, and *IFN-γ*) in the jejunum of broilers. It increased the anti-inflammatory cytokine *IL-10* expression in the jejunum of broilers. EGO also reduced *IL-1β* expression in the lungs, as well as *IL-6* and *TNF-α*, while up-regulating *AvBD5*, *IL-10*, and *IFN-γ* levels. Li et al. (45) demonstrated that 1,8-cineole significantly reduced the levels of TNF-*α* and IFN-*γ* in murine alveolar lavage fluid and lung tissue, while suppressing *NF-κB p65* expression, thereby mitigating lung inflammation. The findings, considered as a whole, indicate that EGO possesses immunomodulatory effects which are not uniform across all tissues; a phenomenon which is potentially indicative of the distinct physiological roles, and immune environments, of the intestinal and respiratory systems. The most intriguing finding pertained to the opposite effect of EGO on *IFN-γ* expression in the jejunum (down-regulated) versus the lung (up-regulated). The significant down-regulation of *IFN-γ* expression in the jejunum observed in this study, combined with previous reports indicating that plant essential oils can reduce pro-inflammatory cytokines (46, 47), suggests that EGO may alleviate intestinal immune stress through its anti-inflammatory activity. In contrast, the elevated levels of *IFN-γ* in lung tissue align with the literature, which indicates that eucalyptus oil stimulates the respiratory immune response in poultry and reduces respiratory infections (48). This suggests that EGO may enhance the body’s respiratory defense capabilities by activating the mucosal immune response in the lungs. These results suggest that EGO exerts anti-inflammatory effects by balancing the expression of pro- and anti-inflammatory cytokines, potentially through modulation of the NF-κB signaling pathway.

Tight junctions (TJ) are critical for maintaining intestinal integrity (49), and their impairment disrupts barrier structure, leading to increased intestinal permeability (50). Mucins (e.g., MUC2, MUC5AC, MUC6) are a class of highly glycosylated glycoproteins secreted by goblet cells (51). In the present study, dietary EGO supplementation exerted tissue-specific effects on barrier-related proteins. In the jejunum, EGO up-regulated TJ proteins (*CLDN1*, *TJP1*, *OCLN*) and the secretory *MUC2*. In the lung, EGO increased *CLDN1*, *TJP1*, and *MUC2* but decreased *OCLN* and *MUC5AC*. The findings indicate that EGO has a positive effect on intestinal barrier function, achieved by means of differential modulation of pulmonary barrier components. The increased expression of *CLDN1*, *TJP1*, and *OCLN* in the jejunum is consistent with previous reports indicating that plant essential oils enhance intestinal barrier function by regulating TJs. Liu et al. (52) showed that carvacrol essential oil up-regulated *OCLN*, *CLDN1*, and *TJP1* gene expression in the small intestine of broiler chickens, findings consistent with ours. Mechanistically, this effect may be mediated through activation of the NF-κB signaling pathway. Sudhoff et al. (53) reported that 1,8-cineole alleviates sinusitis symptoms by suppressing NF-κB activity, reducing the number of mucin-secreting cells, and down-regulating *MUC2* and *MUC19* expression. MUC5AC is the primary secretory mucin in the respiratory tract, secreted by airway epithelial goblet cells. Its overexpression is a key indicator of airway inflammation and hypermucus secretion (54). Therefore, down-regulation of *MUC5AC* typically reflects resolution of airway inflammation and normalization of mucus secretion. Multiple studies support this phenomenon. Lv et al. (55) showed that EGO significantly suppresses the overexpression of *MUC5AC* in a lipopolysaccharide-induced chronic bronchitis model in rats, markedly reducing both the extent and intensity of positive staining areas for mucin expression in tracheal and bronchiolar epithelium. Yu et al. (56) further revealed that eucalyptol not only reduces MUC5AC protein levels but also directly inhibits its mRNA expression, indicating that its regulation occurs at the transcriptional level. An interesting finding in this study is that in the EGO-supplemented group, *OCLN* expression in lung tissue decreased, while *CLDN1* and *TJP1* were significantly up-regulated. This opposite trend, rather than being inconsistent, likely reflects the functional heterogeneity among tight junction components. OCLN is an important tight junction protein primarily found at the tight junctions of epithelial and endothelial cells (57). Shen et al. (58) reported that tight junctions comprise two distinct pathways with different functions: the pore pathway and the leak pathway. The tight junction barrier function is determined by dynamic protein interactions rather than static structures (58). Claudins primarily regulate the pore pathway controlling the permeability of ions and small molecules. In contrast, OCLN is more involved in regulating the macromolecular leak pathway, acting as a dynamic sensor of tight junction integrity (58). Importantly, occludin function is highly dependent on its phosphorylation state. Elias et al. (59) demonstrated that phosphorylation of specific tyrosine residues (Tyr-379 and Tyr-383 in chicken occludin) by c-Src kinase prevents its interaction with ZO-1 and disrupts its proper localization at tight junctions. Therefore, the decreased *OCLN* expression observed in our study may reflect increased phosphorylation and subsequent internalization or degradation, rather than simply reduced synthesis alone. Another intriguing finding of the present study was the opposite expression pattern of *OCLN* in the intestine (up-regulated) versus the lung (down-regulated) following EGO supplementation. First, the expression levels and regulatory patterns of *OCLN* vary across tissues, reflecting the physiological functions of each tissue (60). This tissue-specific response likely reflects the intrinsic heterogeneity of OCLN regulation across different organs. Second, considering the gut-lung axis, the opposite expression patterns may reflect organ crosstalk. In this study, the intestine, as the primary site of EGO exposure, enhances barrier function by up-regulating tight junction proteins (including OCLN, CLDN1, and TJP1), thereby directly initiating a protective response. This improved intestinal barrier may reduce the translocation of pro-inflammatory factors into the circulation, thereby indirectly alleviating pulmonary inflammatory burden (61). Third, *OCLN* expression is controlled by tissue-specific transcription factors. Studies have shown that the OCLN promoter is differentially regulated by specificity protein 3 and yinyang 1 transcription factors in a tissue-dependent manner (62). EGO treatment may modulate the activity or expression of these transcription factors differently in intestinal versus pulmonary tissues, leading to opposite expression outcomes. In summary, these findings provide evidence that dietary EGO supplementation strengthens the intestinal and lung barrier function by modulating TJ protein and mucin expression.

**Figure 5:**
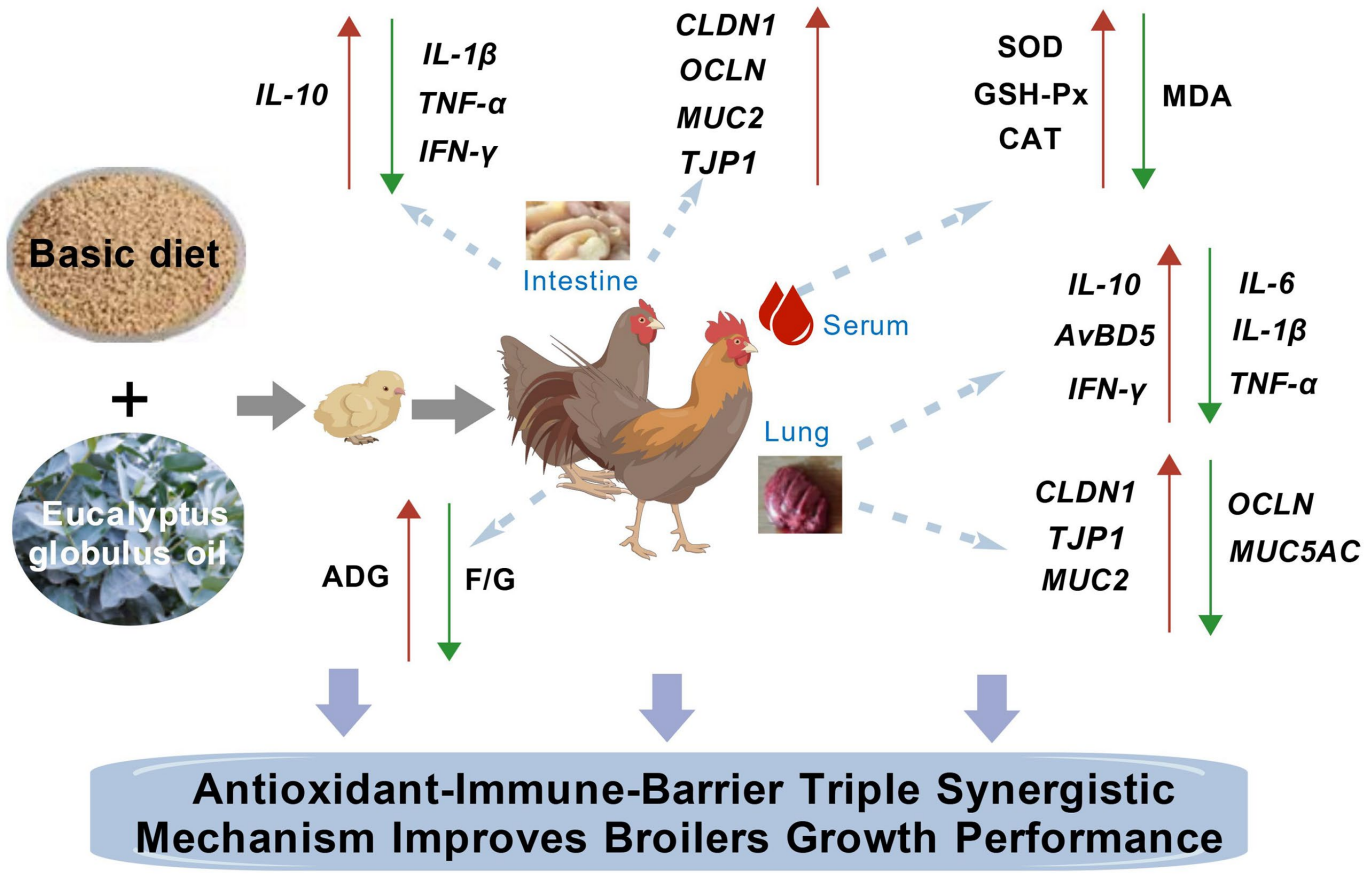
The schematic graphical representation of the effects of *Eucalyptus globulus* oils (EGO) on the growth performance, serum biochemistry, antioxidant status, intestinal health, and lung health of broilers.

Figure 5 illustrates the potential mechanism by which EGO promotes growth in broiler chickens of this study.

## 5 Conclusion

The study demonstrated that dietary supplementation with EGO enhanced growth performance, improved specific serum biochemical parameters, elevated antioxidant capacity, and strengthened immune responses and intestinal and lung tissues of barrier function in broilers. Given growth performance, safety, and efficacy, an additive dosage of 200 mg/kg is recommended.

## Data Availability

The raw data supporting the conclusions of this article will be made available by the authors, without undue reservation.
